# Factors influencing the time to ethics and governance approvals for clinical trials: a retrospective cross-sectional survey

**DOI:** 10.1186/s13063-023-07802-2

**Published:** 2023-12-01

**Authors:** Sam Crosby, Adriana Malavisi, Liping Huang, Stephen Jan, Richard Holden, Bruce Neal

**Affiliations:** 1 The George Institute for Global Health, UNSW Sydney, Sydney, Australia; 2 UNSW Business School, Faculty of Economics, Sydney, Australia; 3 Imperial College London, London, UK

## Abstract

**Background:**

The findings from multi-centre trials are central to the practice of evidence-based medicine, enabling the development and implementation of new treatments. The time it takes to commence clinical trials at sites can be long, and ethics and governance approvals are key steps on the pathway to site activation. The goal of this study was to explore factors influencing the times to ethics approval, governance approval and site activation for multi-centre clinical trials.

**Methods:**

This paper assessed the associations of trial characteristics (disease area and trial phase), site characteristics (government or private ownership, country) and characteristics of the ethics and governance processes (scope guidelines, mutual acceptance requirements and triage of projects by risk) with times to approvals and activation. Median times were compared between site initiations that were and were not exposed to each characteristic using non-parametric tests in univariable and multivariable regressions.

**Results:**

There were data from 150 site activations done across 91 sites, 16 trials and 5 countries from November 2013 to November 2021. The overall median time to activation was 234 days (range 74 to 657), with ethics approval taking a median of 48 days (0 to 369) and governance approval a median of 34 days (0 to 489). Both the univariable and multivariable analyses identified associations of disease area, particularly oncology (*p* univariable = 0.012, *p* multivariable = 0.044), use of scope guidelines (*p* < 0.001, *p* = 0.020) and use of a triage process (*p* < 0.001, 0.043) with shorter median times for governance approval. These characteristics (all *p* < 0.001) plus early trial phase (*p* = 0.028) were also predictive of shorter median times for ethics approval in univariable analyses, but none remained predictive in multivariable models (all *p* > 0.054). The only factors associated with reduced overall time to site activation in both univariable and multivariable analyses were the early trial phase (*p* < 0.001, *p* = 0.013) and mutual acceptance of ethics approvals (*p* = 0.031, *p* = 0.030).

**Interpretation:**

Times to ethics and governance approvals were only one third of total trial start-up time. Factors influencing times to approval and activation were somewhat inconsistent across analyses, but it seems likely that the introduction of selected governance and ethics processes can reduce approval times.

**Supplementary Information:**

The online version contains supplementary material available at 10.1186/s13063-023-07802-2.

## Introduction

Multi-centre randomised clinical trials are gold standard research investigations used to generate high-quality data about ways to prevent, detect or treat medical conditions [1]. The evidence produced by clinical trials forms the basis for the development and implementation of new health interventions, procedures, and technologies, as well as clinical guidelines and government policy. In recent times industrialisation of the health research industry has seen clinical trials become important sources of employment and income generation for individuals, corporations, and government entities as the market size is projected to approach $70 Billion USD by 2028 [2]. Streamlined processes are now central to the clinical, commercial and policy success of multi-centre trials.

In conjunction with pressures for the rapid commencement of clinical trials is the need for oversight and regulation that can ensure the ethics and quality of the projects. Some serious breaches of medical ethics in research done during the last century have highlighted the need for careful ethical review [3]. In parallel, the ethics panels and clinical trial sites responsible for the conduct of clinical trials have identified the need for better-coordinated planning relating to governing how the research will be done in their organisations. For many decades, most jurisdictions doing clinical trials have required an ethics review to protect the dignity, rights, and welfare of research participants as well as a separate site governance review that defines how the research will be implemented at each particular site [4]. Site governance reviews can involve fee schedules, data agreements and other issues specific to that particular site. These two types of reviews are normally performed sequentially with the ethics review going first, but in some cases are considered concurrently.

The ethics and governance infrastructure supporting clinical research has struggled to keep up with the numbers and complexity of clinical trials [5]. For example, large-scale, multi-centre, international trials with pragmatic designs are mostly still required to operate using highly localised ethics and governance requirements with variable approval processes, fees, and timelines [6]. The piecemeal fashion in which the clinical trial sector has evolved in most jurisdictions has meant that the responsibilities of each component of review are often poorly defined. Processes can be overlapping and bureaucratic requiring reduplication of effort, enormous resource, and extended timelines [7]. These delays result in additional costs to researchers which impact that jurisdiction’s competitiveness and attractiveness as a research site [8].

As a response to these challenges, many governments, hospitals, and clinical sites have developed processes that are designed to improve the speed and efficiency of clinical trial applications. A recent systematic review identified almost 100 reports of interventions targeting different aspects of clinical trial administration including 45 targeting ethics approval processes or governance arrangements [9]. Amongst these interventions, the review identified ‘scope guidelines’ (that limit ambiguities in the process and fix timelines), ‘streamlined approval’ (that categorise submissions by risk and triage their review), and ‘mutual recognition’ (where ethics committees acknowledged other committees prior reviews) as showing promise for improving ethics review processes. Scope guidelines, streamlined approval, and ‘coordinating bodies’ (teams employed by trial sites who would actively assist applicants) were identified as having potential for enhancing governance processes.

Clinical Research Organisations do trials in multiple sites across different jurisdictions and often use a clinical trial management system to record standard data about the passage of a trial through the various parts of the regulatory and review processes. In conjunction with data that describe the regulatory environment at each site, this provides an opportunity to explore objectively the association of different regulatory set-ups on the passage of clinical trials through the approval process.

## Methods

This was a retrospective cross-sectional survey of the association between characteristics of the regulatory environment and the times to site activation, ethics approval and governance approval for multi-centre clinical trials. The project received ethics approval from the UNSW Sydney Human Research Ethics Committee on the 29th of March 2021. The STROBE checklist was used to standardize this report [10]. The guiding question for the study was “what are the factors associated with site initiation times and how large are their effects?”.

### Included studies

This study used a convenience sample of trial initiations obtained from the database (GrantPlan) of a collaborating Clinical Research Organisation, George Clinical. A primary search of the company records identified a large number of potentially eligible site initiations, though it was recognised from the outset that incompleteness of information in the GrantPlan database, and limited resource available to ascertain missing data from other sources, would limit the final number of site initiations included in the analysis. Data required for eligibility were disease area, trial phase, and country as well as the dates of initial site contact, ethics submission and approval, governance submission and approval, as well as the date of site activation or first patient recruited.

### Data extraction

George Clinical team members extracted standard data from the clinical trial management database (GrantPlan), the contracts database and other data repositories held by the company between July 2021 and September 2022. The data were provided to a researcher external to George Clinical who de-identified the information by replacing the project name, principal investigator name and site name with unique identification numbers. The standard set of variables were extracted into an Excel database for each trial [11]. Two researchers also examined the websites, contract templates and governance agreement templates of each trial site to collect standard information about the ethics and governance approval processes applicable to each trial. This information was either publicly available on the trial site’s website or available upon request from their research office. This was done in duplicate and independently for the first half of the sites but since there was a very high alignment of findings was done by just one researcher for the remainder of the sites.

### Factors that might influence approval times

The factors that were considered as potential determinants of site activation time fell into three categories—trial characteristics, site characteristics and characteristics of the ethics and governance processes. The latter variously operated at national, sub-national or site levels. The selection of exposures for evaluation was based on two systematic reviews that assessed factors previously reported to influence time to trial start-up [9, 12]. Site initiations were also grouped into pre-2020 and post-2020 groups to explore the impact of COVID-19.

The key trial characteristics considered were the disease area (nephrology; oncology; neurology; endocrinology; paediatric) and the trial phase (1, 2, 3 or 4). The site characteristics recorded were the ownership status (government or private) and the country (Australia, Hong Kong SAR, South Korea, New Zealand, and Taiwan). Researchers were unable to find the ownership status of many trial sites for the Asian countries. The exposures relating to the ethics review process were the use of scope guidelines by the ethics committee; a requirement for mutual acceptance of other ethics committee findings; and an ethics process that triaged applications as low, medium, or high risk. The exposures relating to the governance review were the use of scope guidelines by the governance body; and a governance approval process that triaged applications as low, medium, or high risk.

### Outcomes

An outcome was the time taken to achieve site activation defined as the date that ‘site activation’ was recorded in the clinical trial management system. The date of initial site contact made by the clinical research organisation was used as the date at which the trial start-up process commenced. For 19 trials without a documented ‘site activation’ date, the date of site activation was imputed as the date the first patient was recruited. Additional outcomes were the time from submitting to obtaining ethics approval and the time from submitting to attaining governance approval. The unit of measurement for time was days.

### Analysis

The primary analysis was based on data from 150 site activations including 19 for which missing ‘site activation’ dates were imputed as the date of first patient recruitment. Since the distributions of the times across the included trials were noted to be substantively right-skewed, summary data for site activation, ethics approval and governance approval were reported as medians and ranges. Overall median times and ranges were first summarised for each outcome with all data available and then summarised separately for each subgroup for factors of interest. Differences in the median times across each subset for factors of interest were tested first using univariable regressions and non-parametric tests (Kruskal–Wallis rank sum tests or Wilcoxon rank sum tests) on log-transformed outcome data. We then repeated the analyses using multiple regression methods including all the variables from the univariable analysis, to assess the joint effects of the exposures. Records with missing or unknown values were excluded from relevant analyses with, for example, analysis of the effects of government versus private ownership restricted to Australia because this information could not be obtained for overseas sites. For the analyses of factors influencing time to site activation, a subsidiary analysis was done including only the 131 trials for which a date of site activation was recorded, without the 19 trials with imputed data (Supplementary Table 1). The data was also divided depending upon the year at which trial start-up was commenced (Jan 2014–Dec 2019 versus Jan 2020–Dec 2022) to test for effects of the COVID-19 pandemic. *P*-values < 0.05 were considered statistically significant. All analyses were done using R version 4.1.0 and RStudio 2022.07.2 Build 576 [13, 14]. Packages “gtsummary” and “ggplot2” were used for summarizing the data [15, 16].

## Results

There were 3401 potentially eligible site initiations recorded in the database of George clinical but the great majority (3251, 95%) were excluded because required information was not immediately available in the GrantPlan database and there was no resource available to extract and compile information from other data repositories held by the company. Ultimately there were 150 instances of site activation done at 91 different sites in 5 different countries available for analysis (Table 1). Time to ‘site activation’ was available for 131 clinical trial initiations with data imputed for the other 19 instances using the date of ‘first patient recruited’. Time to ethics committee approval was available for 150 and time to governance approval was available for 145. The most frequent disease area was nephrology (36%) followed by oncology (27%), endocrinology (16%), neurology (15%) and paediatrics (6%). Phase 3 trials were the most common (65%) followed by phase 2 (18%) and phase 1 (11%) and the remainder were registry studies. The data were drawn predominantly from government institutions (72%). There were 84% of site activations done in Australia with the remainder done in Hong Kong SAR (3%), South Korea (7%), New Zealand (3%), and Taiwan (3%). Overall, there were 73% of site activations exposed to 1 or more forms of ethics interventions and 75% exposed to one or more forms of governance intervention. The most frequently applied ethics intervention was triaging of studies into low, medium, or high-risk applications (64%), and this was also the most frequently applied governance intervention (74%). The majority of the site activations commenced before COVID-19 emerged (76%) with the remainder done during or after the pandemic.

**Table 1:** Characteristics of 150 trial start-ups for the 16 multi-centre trials evaluated

	**Number of start-ups (*n* = 150)**	**Frequency**
**Phase**		
1	17	11%
2	27	18%
3	98	65%
4	8	5%
**Disease area of trial**		
Nephrology	54	36%
Oncology	40	27%
Endocrinology	24	16%
Neurology	22	15%
Paediatrics	10	7%
**Country**		
Australia	126	84%
South Korea	10	7%
Hong Kong SAR	5	3%
Taiwan	5	3%
New Zealand	4	3%
**Trial site ownership**		
Government	108	72%
Private	18	12%
Unknown	24	16%
**Scope guidelines for the ethics review**		
Yes	116	77%
No	34	23%
**Mutual acceptance of other ethics committee approval**		
Yes	79	53%
No	55	37%
Unknown	16	11%
**Applications triaged low/medium/high risk for ethics review**		
Yes	96	64%
No	49	33%
Unknown	5	3%
**Scope guidelines used for the governance review**		
Yes	112	75%
No	38	25%
**Applications triaged low/medium/high risk for governance review**		
Yes	111	74%
No	31	21%
Unknown	8	5%
**COVID-19 pandemic**		
Pre-pandemic (Jan 2014–Dec 2019)	114	76%
During or after pandemic (Jan 2020–Dec 2022)	36	24%

### Time to site activation

**Fig. 1:**
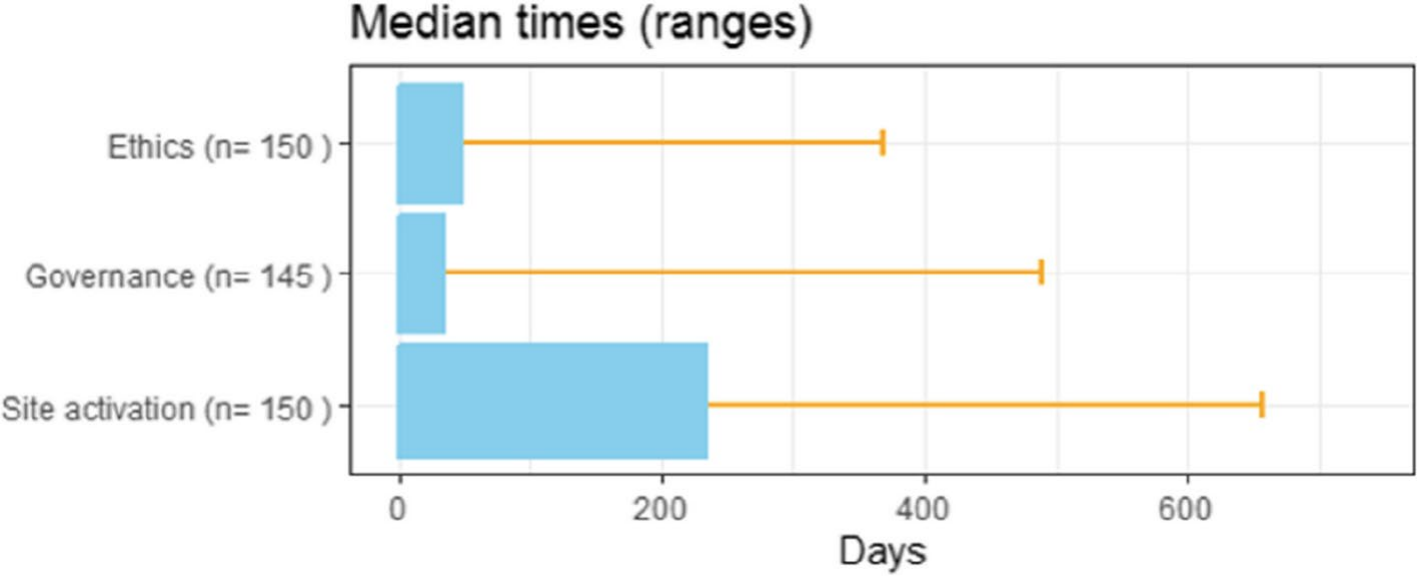
Overall median (range) times for site activation, ethics approval and governance approval. There were 19 site activation times where the date of ‘site activation’ was not recorded and was imputed as the date ‘first patient recruited’

The overall median time to site activation was 234 days with a range that extended from 74 to 657 days (Fig. 1 and Table 2). In the univariable analyses, shorter time to site activations was associated with earlier phase trials (*p* = 0.001), the country in which the trial was done (*p* = 0.039), private ownership of the centre conducting the trial (*p* = 0.039), provisions to allow for the mutual acceptance of other ethics committee approval (*p* = 0.031) and initiation during or after the COVID-19 pandemic (*p* = 0.007). In the multivariable analysis, these associations persisted for only earlier trial phase (*p* = 0.013) and the presence of mutual acceptance of ethics provisions (*p* = 0.030). Disease area was significantly associated with time to site activation in the multivariable analyses alone (*p* = 0.026) with a longer median duration for neurology trials.

**Table 2:** Factors influencing time to site activation (days) for 150 site initiations^a^

	**Median (range)**	Univariable *p*-value* Multivariable p-value**
**Overall** (*n* = 150)	234 (74, 657)	
**Phase**		
1 (*n* = 17)	160 (114, 269)	< 0.001*
2 (*n* = 27)	192 (74, 657)	0.013**
3 (*n* = 98)	250 (106, 617)	
4 (*n* = 8)	289 (85, 350)	
**Disease area of trial**		
Nephrology (*n* = 54)	230 (74, 617)	0.7*
Oncology (*n* = 40)	249 (94, 346)	0.026**
Endocrinology (*n* = 24)	244 (106, 372)	
Neurology (*n* = 22)	294 (82, 657)	
Paediatrics (*n* = 10)	226 (138, 519)	
**Country**		
Australia (*n* = 126)	236 (74, 657)	0.039*
South Korea (*n* = 10)	230 (211, 312)	NA**
Hong Kong SAR (*n* = 5)	395 (262, 426)	
Taiwan (*n* = 5)	174 (154, 252)	
New Zealand (*n* = 4)	184 (177, 196)	
**Trial site ownership**		
Government (*n* = 108)	248 (74, 657)	0.039*
Private (*n* = 18)	188 (114, 484)	0.8**
N/A (*n* = 24)	230 (154, 426)	
**Mutual acceptance of other ethics committee approvals**		
Yes (*n* = 79)	230 (82, 601)	0.031*
No (*n* = 55)	236 (74, 657)	0.030**
Unknown (*n* = 16)	248 (94, 346)	
**Triage by low, medium, or high risk for ethics review**		
Yes (*n* = 96)	250 (74, 657)	> 0.9*
No (*n* = 49)	232 (138, 484)	0.3**
Unknown (*n* = 5)	150 (94, 224)	
**Triage by low, medium, or high risk for governance review**		
Yes (*n* = 111)	236 (74, 617)	> 0.9*
No (*n* = 31)	224 (94, 657)	0.8**
**Scope guidelines for the ethics review**		
Yes (*n* = 116)	233 (74, 657)	0.2*
No (*n* = 34)	235 (144, 484)	0.6**
Unknown (*n* = 8)	178 (114, 252)	
**Scope guidelines for the governance review**		
Yes (*n* = 112)	234 (74, 617)	> 0.9*
No (*n* = 38)	247 (114, 657)	> 0.3**
**COVID-19 pandemic**		
Pre-pandemic (*n* = 114)	248 (82, 657)	0.007*
During or after pandemic (*n* = 36)	192 (74, 617)	0.9**

*NA* not applicable because only one country (Australia) had data to enable multivariable analyses

^*^p-value from univariable comparison

^**^p-value from multivariable comparison that included disease area; phase; ownership status (N/A data removed from ownership status); scope guidelines by the ethics committee; mutual acceptance of other ethics committee findings; an ethics process that triaged applications as low, medium, or high risk; the use of scope guidelines by the governance body; a governance approval process that triaged applications as low, medium or high risk; and pre- or post-COVID in the regression model

^a^Including 19 initiation times imputed as ‘site activation’ as the date the first patient was recruited

Repeating the analysis without the 19 site initiations for which site activation date was imputed (Supplementary Table 1) showed broadly comparable results except that provisions to allow for the mutual acceptance of other ethics committee approval was non-significant in the multivariable analyses, and neurology trials switch from having the longest to the shortest site activation times.

### Time to ethics approval

The median time to ethics approval from ethics submission was 48 days (range 0 to 369 days) (Fig. 1, Table 3) with the 0-day approval times reflecting the impact of mutual acceptance schemes on 2 trial initiations. In the univariable analyses, shorter time to ethics approval was associated with the early trial phase (*p* = 0.028), disease area (particularly oncology) (*p* < 0.001), the use of scope guidelines (*p* < 0.001 univariable), mutual acceptance provisions (*p* < 0.001 univariable) and triaging according to risk (*p* < 0.001 univariable). None of these findings persisted in the multivariable analyses (all *p* > 0.054).

**Table 3:** Factors influencing median time from ethics submission to ethics approval (days) for 150 site activations

	**Median (range)**	Univariable *p*-value* Multivariable p-value**
**Overall**	48 (0, 369)	
**Phase**		
1 (*n* = 17)	15 (5, 106)	0.028*
2 (*n* = 27)	41 (16, 139)	0.7**
3 (*n* = 98)	61 (0, 369)	
4 (*n* = 8)	49 (2, 49)	
**Disease area of trial**		
Nephrology (*n* = 54)	49 (2, 139)	< 0.001*
Oncology (*n* = 40)	21 (3, 106)	0.054**
Endocrinology (*n* = 24)	97 (0, 203)	
Neurology (*n* = 22)	41 (16, 99)	
Paediatrics (*n* = 10)	96 (20, 369)	
**Country**		
Australia (*n* = 126)	48 (0, 369)	0.094*
South Korea (*n* = 10)	46 (11, 75)	NA**
Hong Kong SAR (*n* = 5)	86 (35, 105)	
Taiwan (*n* = 5)	32 (32, 32)	
New Zealand (*n* = 4)	63 (63, 63)	
**Trial site ownership**		
Government (*n* = 108)	48 (0, 369)	> 0.9*
Private (*n* = 18)	46 (8, 215)	0.2**
Unknown (*n* = 24)	54 (11, 105)	
**Scope guidelines for ethics review**		
Yes (*n* = 116)	38 (0, 369)	< 0.001*
No (*n* = 34)	88 (41, 215)	0.2**
**Mutual acceptance of other ethics committee approvals**		
Yes (*n* = 79)	34 (0, 369)	< 0.001*
No (*n* = 55)	67 (13, 215)	0.65**
Unknown (*n* = 16)	18 (6, 125)	
**Triage by low, medium, or high risk**		
Yes (*n* = 96)	38 (0, 369)	< 0.001*
No (*n* = 49)	67 (3, 215)	0.4**
Unknown (*n* = 5)	31 (15, 99)	
**COVID-19 pandemic**		
Pre-pandemic (*n* = 114)	44 (0, 369)	0.5*
During or after pandemic (*n* = 36)	49 (2, 139)	0.8**

*NA* not applicable because only one country (Australia) had data to enable multivariable analyses

^*^p-value from univariable comparison

^**^p-value from multivariable comparison that included phase; disease area; country; trial site ownership status (N/A data removed from ownership status); scope guidelines by the ethics committee; mutual acceptance of other ethics committee findings; an ethics process that triaged applications as low, medium, or high risk; and pre- or post-COVID in the regression model

### Time to governance approval

The median time to achieve governance approval was 34 days (range 0 to 489) (Fig. 1, Table 4). A shorter time to approval was associated with disease area (particularly oncology) (*p* = 0.012), use of scope guidelines for governance review (*p* < 0.001) and a process for triaging review based on risk (*p* < 0.001). All these findings persisted in the multivariable analysis (all *p* < 0.032). In addition, in the multivariable analysis alone, studies done during or after the COVID-19 pandemic had a longer time to governance approval that did trials approved pre-COVID (*p* = 0.013).

**Table 4:** Factors influencing time to governance submission to governance approval (days) for 145 site activations

	**Median (range)**	Univariable *p*-value* Multivariable p-value**
**Overall**	34 (0, 489)	
**Phase**		
1 (*n* = 17)	39 (0, 89)	0.2*
2 (*n* = 27)	45 (1, 140)	0.014**
3 (*n* = 93)	33 (0, 489)	
4 (*n* = 8)	18 (12, 99)	
**Disease area of trial**		
Nephrology (*n* = 49)	37 (0, 489)	0.012*
Oncology (*n* = 40)	17 (0, 112)	0.044**
Endocrinology (*n* = 24)	41 (3, 192)	
Neurology (*n* = 22)	47 (1, 140)	
Paediatrics (*n* = 10)	40 (21, 433)	
**Country**		
Australia (*n* = 126)	34 (0, 489)	0.094*
South Korea (*n* = 10)	10 (0, 102)	NA**
Hong Kong SAR (*n* = 5)	62 (9, 87)	
Taiwan (*n* = 0)	-	
New Zealand (*n* = 4)	30 (7, 56)	
**Trial site ownership**		
Government (*n* = 108)	35 (0, 489)	0.3*
Private (*n* = 18)	28 (3, 77)	0.032**
Unknown (*n* = 19)	14 (0, 102)	
**Scope guidelines used for the governance review**		
Yes (*n* = 110)	28 (0, 489)	< 0.001*
No (*n* = 35)	79 (3, 135)	0.020**
**Triage by low, medium, or high risk**		
Yes (*n* = 111)	24 (0, 489)	< 0.001*
No (*n* = 31)	81 (29, 135)	0.043**
Unknown (*n* = 3)	10 (6, 53)	
**COVID-19 pandemic**		
Pre-pandemic (*n* = 109)	31 (0, 192)	0.12*
During or after pandemic (*n* = 36)	41 (7, 489)	0.013**

*NA* not applicable because only one country (Australia) had data to enable multivariable analyses

^*^p-value from univariable comparison

^**^p-value from multivariable comparison that included disease area; phase; trial site ownership status (N/A data removed from ownership status); the use of scope guidelines by the governance body; a governance approval process that triaged applications as low, medium, or high risk; and pre- or post-COVID in the regression model

## Discussion

There were multiple associations of the characteristics of trials, sites, and review processes with times to regulatory approval and site activation. Both the univariable and multivariable analyses identified associations of disease area, use of scope guidelines and use of a triage process with shorter median times for governance approval. These characteristics plus early trial phase were also predictive of shorter median times for ethics approval in univariable analyses, but none remained predictive in multivariable models. The only factors associated with reduced overall time to site activation in both univariable and multivariable analyses were early trial phase and mutual acceptance of ethics approvals.

In terms of the process characteristics potentially modifiable by institutions there was evidence that streamlined governance processes could reduce governance approval times. Corresponding evidence for benefits from streamlining ethics processes was more limited though mutual acceptance of ethics review outcomes was the only process intervention associated with reduced overall time to site activation. In general, there was a disconnect with benefits of process interventions for ethics or governance timelines not translating into reduced overall times for site activation. This is likely a consequence of the fairly short median times for achieving governance (median 34 days) and ethics (median 48 days) approvals which constituted only about one third of the much longer median time to site activation (234 days).

Ethics and governance approval times are widely considered to be barriers to rapid trial start-up, but these analyses show that other processes in the pathway to site start-up take much more time to be achieved. So, while enhancing ethics and governance interventions can play a role in speeding site activation, the average impact may be limited. Further work to identify whether it is related factors such as the preparation of applications for ethics and governance reviews, or separate issues such as budget negotiations, contract finalisation and other site processes that accrue most of the additional required to achieve site activation.

The positive association of governance interventions with shorter time to governance approval observed in these analyses supports the implementation of governance processes that define the scope of governance review (resulting in a median difference of 51 days) and triage projects according to risk (resulting in a median difference of 57 days). The alignment of the favourable findings for these interventions in the current analyses with positive findings for these types of interventions in prior reports provides further support for their likely value [9].

The results for ethics interventions on ethics approval times were less compelling with considerable inconsistency across the findings for the univariable and multivariable analyses. The observation that schemes that support mutual acceptance of ethics review were associated with shorter overall time to site activation provides additional support for a likely benefit from this strategy. Prior research has also identified mutual acceptance of ethics review as a priority ethics intervention and the totality of the evidence across this study and prior investigations suggests it is likely to be effective [9, 17]. Based on the current data, there remains greater uncertainty about the value of scope guidelines and triaging of ethics applications though both are inherently appealing.

There were several non-modifiable characteristics of studies, like trial phase and disease area, for which associations with time to approval were also observed. Deeper investigation of the reasons why particular types of projects were associated with shorter approval times may provide insight into ways that approval times and site start-up may be reduced. For example, it may be that some aspect of trial start-up has been optimised by an initiative undertaken in a specific disease area, and this could be generalised to other specialities. The findings regarding the effects of the COVID-19 pandemic on approval times in this study were inconclusive. Anecdotal reports suggest that ethics and governance interventions to achieve rapid approval and start-up of COVID-19 studies dramatically reduced timelines for selected projects, but it is unclear whether these changes provided broader system gains or were at the expense of non-COVID-19 projects [18]. Systematic quantitative assessments are required, and this report does not provide insight into this question.

A key strength of this study was the ability to more directly and objectively quantify the association of potential determinants of time to trial start-up identified in prior qualitative and semi-quantitative studies [9]. The joint use of univariable and multivariable analyses provided in-depth insight into the likely robustness of the findings and the separate assessment of times to governance and ethics approvals, as well as overall time to trial start-up, enabled us to place the findings in a broader context. Key challenges were the relatively small size of the dataset and the incomplete data for some site initiations which limited statistical power and meant that clustering at each site was unobservable due to the low numbers of trials performed at each site. There was some inconsistency in the findings across analyses, including the sensitivity analyses excluding initiations without site activation data, and this may reflect statistical instability consequent upon the small numbers. As only 150 of the 3401 site initiations were analysed, there is the significant potential for selection bias and therefore the impact that this has on the representativeness and generalisability of the findings, as well as the possibility of missing variability within the sample. It is also possible that not all-important characteristics of site initiation processes were captured, and residual confounding is possible.

In conclusion, although ethics and governance reviews do not represent the majority of approval time, these analyses support the introduction of streamlined ethics and governance processes by governments and health departments seeking to reduce the time to clinical trial start-up. The introduction of process enhancements should be accompanied by the collection, analysis, and reporting of meta-data to objectively quantify impact.

### Supplementary Information

**Additional file 1: Supplementary Table 1.** Factors influencing time to trial start-up (days) restricted to 131 site initiations with ‘site activation’ date.

**Additional file 2. **STROBE Statement—checklist of items that should be included in reports of observational studies.

## Data Availability

The datasets used and/or analysed during the current study are available from the corresponding author on reasonable request.
