# Supplementary material for: Factors influencing the time to ethics and governance approvals for clinical trials: a retrospective cross-sectional survey
Source: Trials. 2023 Dec 1;24:779. doi: 10.1186/s13063-023-07802-2 (PMC10693024; doi:10.1186/s13063-023-07802-2)
Supplement: Supplementary file 1 — Additional file 1: Supplementary Table 1. Factors influencing time to trial start-up (days) restricted to 131 site initiations with ‘site activation’ date. [file 13063_2023_7802_MOESM1_ESM.docx]

## Supplementary table 1 Factors influencing time to trial start-up (days) restricted to 131 site initiations with ‘site activation’ date

|  | **Median (range)** | **Univariable p-value ***  **Multivariable p-value**** |
| --- | --- | --- |
| **Overall** | 234 (74, 617) |  |
| **Phase** |  |  |
| 1 (n=11) | 156 (114, 269) | <0.001* |
| 2 (n=16) | 168 (74, 337) | 0.009** |
| 3 (n=96) | 249 (106, 617) |  |
| 4 (n=8) | 289 (85, 350) |  |
| **Disease area of trial** |  |  |
| Nephrology (n=54) | 230 (74, 617) | 0.5* |
| Oncology (n=34) | 270 (94, 346) | 0.049** |
| Endocrinology (n=24) | 244 (106, 372) |  |
| Neurology (n=9) | 191 (82, 601) |  |
| Paediatrics (n=10) | 226 (138, 519) |  |
| **Country** |  |  |
| Australia (n=107) | 235 (74, 617) | 0.028* |
| South Korea (n=10) | 230 (211, 312) | NA** |
| Hong Kong SAR (n=5) | 395 (262, 426) |  |
| Taiwan (n=5) | 174 (154, 252) |  |
| New Zealand (n=4) | 184 (177, 196) |  |
| **Trial site ownership** |  |  |
| Government (n=90) | 248 (74, 617) | 0.06* |
| Private (n=17) | 189 (114, 484) | 0.6** |
| Unknown (n=24) | 230 (154, 426) |  |
| **Scope guidelines used for the ethics review** |  |  |
| Yes (n=97) | 234 (74, 617) | 0.2* |
| No (n=34) | 235 (114, 484) | 0.2** |
| **Scope guidelines used for the governance review** |  |  |
| Yes (n=96) | 234 (74, 617) | 0.8* |
| No (n=35) | 245 (114, 426) | 0.3** |
| **Mutual acceptance of other ethics committee approvals** |  |  |
| Yes (n=67) | 230 (82, 601) | 0.026* |
| No (n=49) | 236 (74, 617) | 0.3** |
| Unknown (n=15) | 249 (94, 346) |  |
| **Triage by low, medium, or high risk for governance review** |  |  |
| Yes (n=95) | 240 (74, 617) | 0.7* |
| No (n=28) | 220 (94, 426) | 0.8** |
| Unknown (n=8) | 178 (114, 252) |  |
| **Triage by low, medium, or high risk for ethics review** |  |  |
| Yes (n=80) | 244 (74, 617) | 0.8* |
| No (n=49) | 232 (138, 484) | 0.4** |
| Unknown (n=2) | 159 (94, 224) |  |
| **COVID-19 pandemic** |  |  |
| Pre-pandemic (n=95) | 249 (82, 601) | 0.007* |
| During or after pandemic (n=36) | 192 (74, 617) | 0.5** |

* p-value from univariable comparison

** p-value from multivariable comparison that included disease area; phase; ownership status (N/A data removed from ownership status); scope guidelines by the ethics committee; mutual acceptance of other ethics committee approvals; an ethics process that triaged applications as low, medium, or high risk; the use of scope guidelines by the governance body; a governance approval process that triaged applications as low, medium or high risk; and pre- or post-COVID in the regression model.

NA=not applicable because only one country (Australia) had data to enable multivariable analyses.
